# Stone Milling versus Roller Milling in Soft Wheat (Part 2): Influence on Nutritional and Technological Quality of Products

**DOI:** 10.3390/foods11030339

**Published:** 2022-01-25

**Authors:** Marina Carcea, Valentina Narducci, Valeria Turfani, Enrico Finotti

**Affiliations:** Research Centre for Food and Nutrition, Council for Agricultural Research and Economics (CREA), Via Ardeatina 546, 00178 Rome, Italy; valentina.narducci@crea.gov.it (V.N.); valeria.turfani@crea.gov.it (V.T.); enrico.finotti@crea.gov.it (E.F.)

**Keywords:** soft wheat, wholegrain flour, stone milling, roller milling, composition, trans-fatty acids, phytic acid, damaged starch

## Abstract

Wholegrain soft wheat flours can be obtained by either roller milling or stone milling. In this paper, we report on the continuation of a study aimed at analysing compositional and technological differences between differently milled wholegrain flours. Eight mixes of soft wheat grains were stone milled and roller milled and the milling products analysed for their phytic acid, lipids composition to determine the presence of trans-fatty acids and damaged starch. A wholegrain flour milled with a laboratory disk mill was also analysed as comparison, as well as seven wholegrain flours purchased on the market. For phytic acid we found that that there is no compositional difference between a stone milled or a roller milled flour if the milling streams are all recombined: the milling streams instead have different amounts of phytic acid which is mainly present in the fine bran and coarse bran. It was not possible to highlight differences in the milling technology due to the presence of trans-fatty acids in the stone milled wholegrain flour whereas it was possible to find that starch damage depended on the milling method with stone milled wholegrain flours having in all cases significantly higher values than the roller milled ones.

## 1. Introduction

Evidence is accumulating in the scientific literature regarding the health benefits of the consumption of wholegrain cereals in the human diet. Epidemiological and intervention studies point in the direction of a reduced risk of cardiovascular diseases, type 2 diabetes, colorectal, pancreatic, and gastric cancers, weight gain, and obesity [1,2,3,4]. Dietary fibre greatly contributes to these benefits but does not explain them all. Although fibre is the most extensively studied, it is only one of a complex array of beneficial bioactive components, like phytochemicals, complex carbohydrates, proteins, minerals, lipids, and vitamins that are present in different proportions in the bran, germ, and endosperm fractions of the whole grain and vary in concentration depending on the cereal species [5]. Previous studies have shown that the bioactive compounds are mainly concentrated in the outer layers of the grain, and that their distribution within the kernel differs according to the class of nutrients [6].

Because of growing scientific evidence, consumption of wholegrain cereals is recommended in the dietary guidelines of many countries [7] and the market for wholegrain ingredients and foods is booming worldwide in consideration also of sustainability issues connected to the consumption of unrefined vegetable foods.

In parallel, the scientific community realized the need to define in clear terms a wholegrain raw material and a wholegrain food product because there are several kinds available to consumers, possessing different compositions and hence different nutritional qualities, even if they are all called “wholegrain” foods. A globally agreed definition of a wholegrain raw material and a wholegrain food is necessary as a basis for dietary recommendations, food labelling, legislation, health claims, and to be able to compare nutrition research where the studied materials should be clearly known. In order to fill this gap, the American Association of Cereal Chemists International (AACCI) and the Healthgrain Forum have published definitions for a wholegrain raw material [8,9]. More recently the Whole Grain Initiative, a global group of experts from academia, government agencies, and industry, developed definitions of wholegrain as a food ingredient and of wholegrain foods to be globally adopted to ensure that all stakeholders have shared standards and to gain the consumers’ trust in what they buy [10]. 

Milling is the first step in the production of a wholegrain flour and it can be done by either roller milling or stone milling. The technological process used in the production of the flour might influence its technological and nutritional qualities, so different wholegrain flours can be obtained from the same raw-material grains [11,12].

Roller milling is the prevalent milling technology: in this process, the external layers of the grain become part of some grinding fractions that are separated from the refined flour. The bran fractions, which also include the germ, can be recombined with the refined flour to give a wholegrain flour. If the recombination happens in different proportions, we can have different wholegrain flours with different contents of compounds potentially useful for human health. Stone mills instead are able to directly produce a wholemeal flour by keeping all the kernels’ components in the flour at all times but they are known to generate considerable heat due to friction that might affect the technological and nutritional quality of the resulting flours [13,14,15]. It follows that wholemeal products, even if obtained from flours of the same botanical species, can be very different from each other in terms of benefits for those who consume them.

Conscious of the fundamental role of the milling technology to determine the nutritional and technological characteristics of a wholegrain flour and considering the paucity of rigorous scientific studies useful for their differentiation and classification, in a previous paper published in 2020 [14] we set out to investigate the quality of stone milled flours and roller milled fractions from the same soft wheat grains, and of stone milled and roller milled wholegrain flours purchased on the market. We determined the content of protein, ash, total lipids, total dietary fibre, total polyphenols, and alkylresorcinols as well as the particle size distribution of the flours. The idea was to contribute compositional data to the current knowledge of the range of nutritional and technological qualities that can be found in a soft wheat wholegrain flour milled in different ways, useful for composition tables, legislators, nutritional studies, the food industry, as well as consumers.

In this paper we want to report on the continuation of our research which went on to study other quality parameters such as phytic acid, damaged starch, and trans-fatty acids in the same samples, to continue assessing differences in wholegrain soft wheat flours due to processing. These two latter parameters could be distinctive of wholegrain flour processing. In fact, it is known that in the roller milling process, the shear and compressive forces on endosperm are minimal, resulting in a lower loss of heat-sensitive components in the flour, whereas in stone mills heat and friction are created. It is therefore thought that this can result in considerable damage to starch, protein, and unsaturated fatty acids in comparison with other milling techniques.

## 2. Materials and Methods

### 2.1. Samples, Milling and Sample Preparation for Analyses

Eight samples of stone milled soft wheat flours (SMF) produced by different mills in central Italy, plus their corresponding clean grains (*Triticum aestivum* L.) before milling, were collected at the manufacturer. The grains were a mix of different varieties, coming from different locations and they represented the raw material commonly used by commercial mills.

A portion of the grains was also ground by means of a laboratory Bühler MLI 204 disc mill (Bühler, Uzwill, Switzerland) to produce a wholegrain flour (WGF) which was used as reference for a perfect wholemeal flour because it surely contained all components in the grain.

Another portion of the same grains was roller milled in our laboratory to obtain three different mill streams, namely refined white flour (RF), coarse bran (CB) and fine bran (FB): these fractions were recombined to obtain a wholegrain roller milled flour (RRMF). 

In addition to the above samples, seven commercial whole wheat flours from six different brands were purchased in stores. Three of them were labelled “stone milled” wholemeal, the other four were “wholemeal” only, certainly roller milled. These commercial flours (C1-C7) were used as comparison.

For roller milling, grains were tempered to 15.5–17.5% moisture (for 36–48 h depending on their hardness measured by means of the SKCS 4100 instrument, Perten Instruments, Stockholm, Sweden) and subsequently milled in a Bühler MLU 202 pilot mill (Bühler, Uzwill, Switzerland) equipped with three break rolls, three reduction rolls and six screens, according to method AACC 26-10.02 [16] with an average of 71% extraction rate for the refined flour. Roller milling of wheat produces various fractions with different physicochemical characteristics: the characteristics of all the roller milling fractions together with those of the stone milled flours are reported in Carcea et al. [14].

Sample preparation for analyses implied that all samples were sifted before analysis and the residue not passing the 494 μm sieve was ground again by the above described Bühler MLI 204 laboratory mill until it all passed through the sieve.

### 2.2. Analyses

Moisture of all flours and roller milled fractions was determined by oven drying to constant weight according to ICC Method N. 110/1 [17].

The lipid fractions of the eight stone milled flours, the eight reconstituted roller milled flours and the eight wholegrain flours used as reference were first obtained by hydrolysis in formic acid and hydrochloric acid at 75 °C reflux for 20 min followed by extraction in hexane and evaporation, according to ICC Method N. 136 for total lipids [17]. Then, trans fatty acids were determined by Fourier Transform Infrared Spectroscopy (FTIR), by means of a Bruker Tensor 27 instrument (Bruker Corporation, Billerica, MA, USA) equipped with Attenuate Total Reflectance (ATR). A drop of oil sample was placed on the diamond crystal plate and the spectrum was recorded in transmittance mode with 16 scans in the spectral range of 400–4000 cm^−1^.

Phytic acid was determined on each one of the five milling products obtained from the eight grain samples and on the seven commercial flours, by a quantitative kit method (K-PHYT, Megazyme Int., Wicklow, Ireland) following the procedure of McKie and McCleary [18]. In this procedure, the sample is extracted with acid and the extract is treated with a specific phytase that hydrolyses phytic acid and lower myo-inositol phosphate forms into myo-inositol monophosphate. Subsequent treatment by alkaline phosphatase releases phosphate (phosphate released by other monophosphate esters is negligible). Finally, phosphate is measured by a colorimetric assay involving reaction with ammonium molybdate followed by reduction to molybdenum blue and reported as total phosphorus or phytic acid content of the original sample.

Damaged starch was measured on the eight stone milled flours and on the eight reconstituted roller milled flours according to AACC Method 76–31.01 [16] using a reagent kit (K-SDAM, Megazyme Int., Wicklow, Ireland). In this procedure, damaged starch granules are hydrated and hydrolysed to maltosaccharides plus α-limit dextrins by carefully controlled treatment with purified fungal α-amylase. This reaction is terminated on addition of diluted sulphuric acid, and aliquots are treated with excess levels of purified amyloglucosidase to give complete degradation of starch-derived dextrins to glucose. The glucose is specifically measured with a high purity glucose oxidase/peroxidase reagent mixture. Values are presented as starch (damaged) as a percentage of flour weight on a dry matter basis.

### 2.3. Chemicals

All solvents were of analytical grade and reagents were of the highest available purity. N-hexane, 96% ethyl alcohol, 99% formic acid, 37% hydrochloric acid, 96% sulphuric acid, glacial acetic acid, calcium chloride dihydrate, anhydrous sodium sulphate, and ammonium molybdate tetrahydrate were purchased from Carlo Erba (Milan, Italy). Ascorbic acid, trichloroacetic acid, and sodium hydroxide were purchased from Sigma (Saint Louis, MO, USA).

### 2.4. Data Presentation and Statistics

Moisture and total lipids were determined in duplicate, damaged starch and phytic acid in triplicate. Mean of independent determinations were calculated, together with standard deviation. Damaged starch and phytic acid are reported on a dry matter basis in Tables and Figures and in the text. Microsoft Excel was used for these calculations. Data were analysed by Student’s *t*-test and one-way ANOVA (factor = milling product, including kind of milling—stone or roller or disc—and roller milling fractions—refined flour, shorts, bran; factor “sample” included milling product and grain origin) followed by Tukey’s honestly significant difference (HSD) test, using the PAST statistical package version 2.17c [19].

## 3. Results

**Figure 1:**
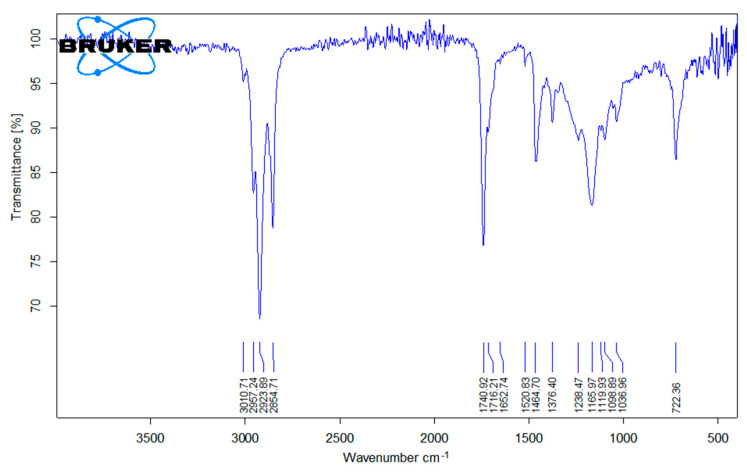
Representative FTIR spectra of lipids extracted from wholegrain stone milled, recombined roller milled, reference flour and commercial wholegrain soft wheat flours.

A representative FTIR spectrum of the lipid fraction extracted from stone milled and reconstituted roller milled flours from the eight grain mixes is presented in Figure 1. Irrespective of the milling method, we obtained nearly identical spectra, representing the pattern of a mixture of lipids in which a great portion is represented by triglycerides and other fractions are phospho- and glycolipids, fatty acids, sterols, mono and diglycerides, hydrocarbons and sterol esters, and where there is a large fraction of unsaturated fatty acids [20,21].

The spectra showed several bands of strong (s), medium (m), or weak (w) intensity, often coalescent and sometimes with visible shoulders. The bands that could be attributed with reasonable certitude are pointed out in Figure 1 and are illustrated hereafter. In the frequency range of alkane and alkene carbon-hydrogen bond stretching: 3009 cm^−^^1^, weak (stretching of alkenic C-H bonds in the fatty acid chains); 2956 cm^−^^1^, medium (asymmetric stretching of C-H bonds in CH_3_ groups); 2924 cm^−^^1^, strong (asymmetric stretching of C-H bonds in CH_2_ groups); 2879 cm^−^^1^, shoulder medium (symmetric stretching of C-H bonds in CH_3_ groups); 2954 cm^−^^1^, medium-strong (symmetric stretching of C-H bonds in CH_2_ groups). In the frequency range of carbonyls: 1739 cm^−^^1^, strong (stretching of the double C=O bonds in ester groups); this band presented two shoulders at 1714 cm^−^^1^ (m) and 1690 cm^−^^1^ (w). In the frequency ranges of bending of alkane carbon-hydrogen bonds: 1462, medium (symmetric scissoring of CH_2_ groups coalesced with asymmetric scissoring of CH_3_ groups); 1375 (symmetric scissoring of CH_3_); 722, m (rocking of CH_2_). In the range of alcoholic carbon-oxygen bonds: a zone of absorbance between 1300 and 1000 with a strong band centred at 1168 cm^−^^1^ attributable to the stretching of alcoholic C-O bonds of ester groups. Bands attributable to the stretching of carboxylic hydrogen-oxygen bonds of free fatty acids (free or engaged in hydrogen bonding) were not visible in the spectra. Trans mono-alkene fatty acids in lipids have a diagnostic band at 966 cm^−^^1^, due to the out-of-plane bending of the C-H alkene bonds, that is proportional to the trans fatty acid content and that is used for trans fatty acid quantitation (up to 1%) by the industry for regulatory compliance [22]. In our case, the spectral region around 966 cm^−^^1^ (precisely below 1000 cm^−^^1^ down to 900 cm^−^^1^) presented weak bands, none of which was at 966, changing from sample to sample. Differences between the spectra of flours from the same grain mix but milled in different ways, i.e., roller milling or stone milling, could not be evidenced.

**Figure 2:**
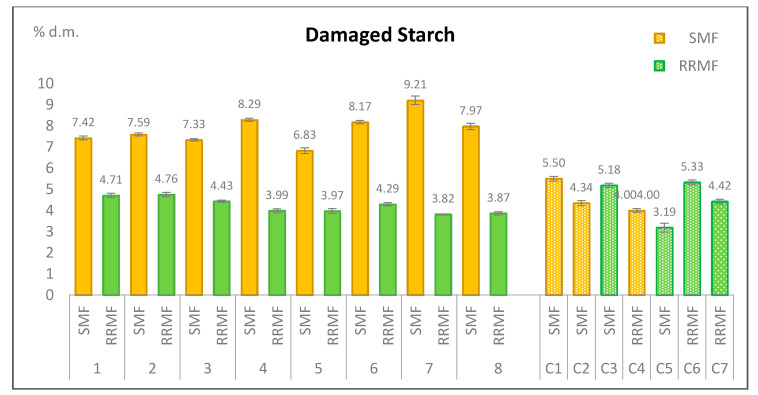
Damaged starch content in wholegrain stone milled, recombined roller milled and commercial soft wheat flours.

Figure 2 shows the damaged starch (DS) content of the eight stone milled flour samples (SMF), of the eight reconstituted roller milled flour samples (RRMF), toghether with that of the seven commercial wholemeal flours (C) of which three were stone milled and four were roller milled. Starch damage was found to depend on the milling method. In fact, stone milled flours contained from 6.83 to 9.21% d.m. of damaged starch, significantly (*p* < 0.01) more than reconstituted roller milled flours that contained from 3.82 to 4.76% d.m. of it. The two ranges of values are completely separated and values in stone milled and roller milled flours from the same grain mix differed more than three times the sum of the standard deviations, for all the eight grain mixes. However, if we consider the starch damage of the seven commercial flours purchased in stores, the three stone milled ones had 4.00–5.50% d.m. and the four roller milled ones had 3.19–5.33% d.m. of damaged starch. In this case, the two ranges of values partially overlap and the two groups of samples are not significantly different (*p* < 0.05), even if the highest starch damage was found in stone milled flours and the lowest in roller milled flours. Starch damage in commercial flours (stone or roller miled) was substantially similar (*p* < 0.05) also to that of our eight roller milled flours (*p* < 0.05). On the contrary, our eight stone milled flours contained significantly (*p* < 0.01) more damaged starch than commercial flours, both stone and roller milled.

Table 1 illustrates the phytic acid content of the eight stone milled flours, the eight reconstituted roller milled flours, the eight reference wholemeal flours and the 3 × 8 roller milling fractions from the eight grain mixes. The stone milled flours contained from 1.00 to 1.21 g/100 g d.m. of phytic acid, the reference disc-milled wholegrain flours from 0.96 to 1.31 g/100 g d.m. and the reconstituted roller milled flours from 0.95 to 1.62 (for RRMF, values were calculated from data on milling fractions and milling yield of each fraction). These three kinds of flour contain substantially all the phytic acid present in the kernel (*p* < 0.05), that was globally around 1–1.6 g/100 g d.m.

Analysis of the roller milling fraction evidenced the inhomogeneous distribution of phytic acid in the grains: refined flour contained from 0.10 to 0.17 g/100 g d.m., i.e., only 9–15% the content of the entire grain, and phytic acid was concentrated in fine bran (from 2.28 to 2.58 g/100 g d.m., i.e., from 180 to 250% the content of the whole kernel) and even more in coarse bran (from 3.66 to 5.42 g/100 g d.m., i.e., from 321 to 534% the content of the whole kernel). Significant differences were due to the presence or not of all kernel components in the milling product and not to the milling method. The phytic content of the commercial flours is shown in Table 2. These flours, which were all labelled as wholegrain, had a phytic acid content ranging from 0.67 to 1.02 g/100 g d.m. for the stone milled ones and from 0.73 to 0.94 for the other ones. From a statistic point of view, phytic acid in commercial flours did not depend on milling type (*p* < 0.05). Commercial flours, instead, had globally a lower phytic acid content than the eight grain mixes (*p* < 0.05).

## 4. Discussion

The FTIR spectra, in the conditions of our analysis, didn’t reveal the formation of trans fatty acid in detectable amounts either with stone milling or roller milling. Prabashankar and Rao [23] reported that stone milling, due to the higher heat generated by friction, induced degradation of fatty acids, particularly a decrease in linoleic and an increase in palmitic acid, possible due to reduction of unsaturated fatty acids to saturated. This had prompted us to check if trans-fatty acid could be formed in analogous conditions. However, in our case the lipid fractions from the different milling methods gave substantially the same spectra, without evident signals of trans fatty acids. It must be said that we don’t know the temperatures that were reached in the eight industrial stone mills that prepared the flours we analysed, that several factors influence the milling performance [13] but also that modern technologies might allow for a better setting of stone milling conditions.

The phytic acid data show that milling per se doesn’t influence the phytic acid content in the flour, unless the external layers of the grains are removed: it is refinement, rather than heat due to friction, to affect phytic acid content in flours. It is notable that phytic acid in our eight refined flour samples was 9–15% the content of the entire kernel and that the ranges of values found for wholegrain flour and refined flour from the two milling methods were sharply separated (around 1.0–1.6 g/100 g d.m. for wholegrain flour and 0.10 to 0.17 g/100 g d.m for refined flour). This could be of help in updating requirements for allowing a flour to be denominated wholemeal. It is also worth mentioning that phytic acid is present at the highest levels in the bran and still in important amounts in the fine bran where it is known to be an antinutritional compound due to its ability to bind to minerals, proteins and starch, limiting their bioavailability: however, also its beneficial activity is being studied and the prevention of certain cancers, heart diseases, renal stones, and chemotherapy side effects are attributed to its antioxidative effects and metal binding capacity [24].

Damaged starch refers to the amount of grain starch granules physically broken or fragmented during milling and depends on kernel hardness and grinding intensity. It is a quality parameter for a wheat flour because damaged starch is more susceptible to enzymatic hydrolysis, it influences the water absorption capacity of the flour, dough stickiness, bread crust colour and for leavened products, yeast activity during fermentation [25]. The optimum damaged starch value varies with the use of the flour and the type of bread. Milling produces the damaged starch so the milling technique significantly influences the flour performance during dough making and the bread characteristics: different results can be achieved by choosing different settings in the mill [11,26]. Our results also confirm that starch damage depends on the kind of milling and under the controlled milling conditions of our experiment, stone milled flours always gave a higher damaged starch content than recombined roller milled flour. However, appropriate choices of milling settings and post-milling treatments can reduce this difference and thus commercial flours sold in stores, milled with different techniques, can have approximately the same range of starch damage values. Sifting after milling to comply with specific law requirements which set limits for certain parameters such as ash, still in place in several countries for soft wheat wholegrain flour (for example in Italy, ash content must be between 1.30 and 1.70 for 100 g d.m.), can affect both the phytic acid and the starch damage content. Many authors report that starch damage is more pronounced in stone milled flours due to the higher temperatures generated during stone milling (60–90 °C versus 35–40 °C in roller milling) [13,23,27,28]. Besides, Liu et al. [29] found that damaged starch content of wheat flour increased significantly with the decrease of particle size. On the contrary, Kihlberg et al. [30] found that damaged starch content was higher for roller-milled samples than for the stone milled. These different conclusions can be related to the varietal composition of the original grains from which flours were milled, but also to the different mills and their settings such as wheat tempering, feed rate, aperture, rotational speed, abrasiveness and sifting [13,26,27,31].

## 5. Conclusions

In this paper we completed the study of a group of quality parameters that we had set as potential indicators of differently milled wholegrain flours by analyzing phytic acid, trans-fatty acids, and damaged starch. In the recent review by Cappelli et al. [11] they concluded that only a few studies have specifically compared stone milling and roller milling techniques and pointed out the need for extensive comparative studies of their different effects: our two papers are an attempt to fill this gap.

In our previous paper we concluded that stone milling produces a wholegrain flour where all kernel components are present, however, if in roller milling all the streams are recombined in the same proportions as in the original grains, there are no compositional differences between the two kinds of flour. The different fractions in roller milling have different compositions and can be used on their own.

In this paper we demonstrated that for phytic acid also significant differences between the two kinds of flours were due to the presence or not of all kernel components in the milling product, and not to the milling method. Under the conditions of our study where lipids were studied by means of the FTIR, we were also not able to demonstrate differences in the milling technology due to the presence of trans-fatty acids in the stone milled flour spectra. In accordance with our previous finding on particle size distribution, we found instead that starch damage depended on the milling method with stone milled flours having in all cases significantly higher values than the roller milled ones. Particle size distribution and damaged starch can both have an impact on the technological and the nutritional quality of flours. However, we also found that processing procedures subsequent to milling, such as sifting, can have an effect on damaged starch values.

## Figures and Tables

**Table 1:** Phytic acid in milling products obtained by means of stone and roller milling (and reference flour) from eight soft wheat grains.

Grain Code	Milling Product	Phytic Acid (g/100 g d.m.)	#	§	Grain Code	Milling Product	Phytic Acid (g/100 g d.m.)	#	§
1	WGF	1.01	*c*	*j*	5	WGF	1.13	*c*	*ij*
	SMF	1.13	*c*	*ij*		SMF	1.07	*c*	*ij*
	RF	0.10	*d*	*k*		RF	0.14	*d*	*k*
	FB	2.58	*b*	*fg*		FB	2.39	*b*	*gh*
	CB	4.99	*a*	*bc*		CB	4.38	*a*	*d*
	RRMF	1.01				RRMF	1.17		
2	WGF	1.05	*c*	*ij*	6	WGF	1.11	*c*	*ij*
	SMF	1.13	*c*	*ij*		SMF	1.12	*c*	*ij*
	RF	0.14	*d*	*k*		RF	0.11	*d*	*k*
	FB	2.74	*b*	*f*		FB	2.28	*b*	*h*
	CB	5.42	*a*	*a*		CB	5.42	*a*	*a*
	RRMF	0.99				RRMF	1.19		
3	WGF	0.96	*c*	*j*	7	WGF	1.31	*c*	*i*
	SMF	1.00	*c*	*j*		SMF	1.21	*c*	*ij*
	RF	0.10	*d*	*k*		RF	0.13	*d*	*k*
	FB	2.33	*b*	*gh*		FB	2.39	*b*	*gh*
	CB	5.13	*a*	*b*		CB	4.77	*a*	*c*
	RRMF	0.95				RRMF	1.26		
4	WGF	1.14	*c*	*ij*	8	WGF	1.29	*c*	*i*
	SMF	1.01	*c*	*ij*		SMF	1.17	*c*	*ij*
	RF	0.17	*d*	*k*		RF	0.12	*d*	*k*
	FB	2.38	*b*	*gh*		FB	2.47	*b*	*fgh*
	CB	3.66	*a*	*e*		CB	4.79	*a*	*c*
	RRMF	1.04				RRMF	1.62		

d.m. on dry matter; WGF wholegrain flour from Bühler MLI 204 laboratory disc mill, as reference. Roller milling fractions: RF refined flour, FB fine bran, CB coarse bran; RRMF recombined roller milled flour; SMF stone milled flour; # ranking after ANOVA amongst different milling products from the same grain mix (*p* < 0.01); § ranking after ANOVA amongst all WGF, SMS, RF, FB and CB samples in the Table (*p* < 0.05); RRMF samples not included in ANOVA because they are calculated values.

**Table 2:** Phytic acid in commercial soft wheat wholegrain flours.

Product Code	Milling Product	Phytic Acid (g/100 g d.m.)	#
C1	SMF	0.90	*b*
C2	SMF	0.88	*b*
C3	SMF	1.02	*a*
C4	RRMF	0.94	*ab*
C5	SMF	0.67	*c*
C6	RRMF	0.73	*c*
C7	RRMF	0.91	*b*

d.m. on dry matter; C commercial flour; SMF stone milled flour; RRMF recombined roller milled flour; # ranking after ANOVA (*p* < 0.05).

## Data Availability

The data presented in this study are available on request from the corresponding author.
